# Supplementary material for: Origin and evolutionary malleability of T cell receptor α diversity
Source: Nature. 2023 Jun 21;619(7968):193–200. doi: 10.1038/s41586-023-06218-x (PMC10322711; doi:10.1038/s41586-023-06218-x)
Supplement: Supplementary file 1 — Evolutionary origin of adaptive immune receptors; selection of a target antigen receptor locus for phylogenetic analysis; the role of TdT in diversification of CDR3 regions. Supplementary Figs. 1–7 and refs. 71–76. [file 41586_2023_6218_MOESM1_ESM.pdf]

nature portfolio

https://doi.org/10.1038/s41586-023-06218-x

Supplementary information

Origin and evolutionary malleability of T cell receptor α diversity

In the format provided by the authors and unedited

Supplementary Text

Evolutionary origin of adaptive immune receptors

Innate immune systems rely on germline-encoded receptors for self/nonself discrimination. Natural selection has shaped the specificity of these receptors in such a way that they are broadly tuned to group-specific molecular patterns of pathogens, such as bacterial cell wall components, but have minimal or no reactivity towards self-structures. By contrast, adaptive immune systems rely on somatically diversified antigen receptors to discriminate among closely related molecular structures, such as peptides differing by a single residue in the case of TCRs, or stereoisomers in the case of antibodies. Since extant vertebrate species generate astoundingly large repertoires of different antigen receptors, much effort is devoted to identifying the various layers of quality control mechanisms with which animals eliminate or suppress self-reactive lymphocyte clones. But how, after the sudden emergence of somatic recombination, did ancient vertebrates avoid potentially harmful self-reactivity emanating from somatically assembled receptors, when such quality controls were probably not yet in place?

A possible solution to this conundrum is proposed here. It is based on one of the central predictions of the transposon/split gene hypothesis[17]. When the proto-variable (V) and proto-joining (J) segment was formed by insertion of a transposon-like element into an exon of a gene encoding a cell surface receptor, both V and J elements became flanked by a short identical sequence, the target-site duplication (TSD)[18,21,43]. We hypothesize that the TSD served as a microhomology region, minimizing – at least initially – sequence variation at the junction after re-assembly of the V and J parts of the split receptor exon by the NHEJ repair process. Hence, we suggest that the extent of diversity of the somatically assembled receptors was initially small, and consider the possibility that the primordial type of AgRs had innate-like properties, perhaps tuned to more general properties of potential non-self/pathogen-derived antigens. In the general context of adaptive immunity, it is conceivable that the proliferative property of 'adaptive' lymphocytes constituted the initial advantage over the more stereotyped response of innate effector cells. Selection pressure for fine-grained discrimination of antigenic structures may have resulted in the degradation of microhomology regions, and the recruitment of non-template-directed DNA polymerases, such as terminal deoxynucleotidyl transferase (TdT) (see below) to diversify the CDR3 regions of AgRs. To avoid undesired self-reactivity, the capacity to more efficiently diversify antigen binding surfaces must have evolved in lockstep with the emergence of suitable central and peripheral quality control mechanisms to curb undesired self-reactivity.

Selection of a target AgR locus for phylogenetic analysis

In extant jawed vertebrates, the presumed primordial configuration of the split V-J exon[1,17] survived in *TRA*, *TRG*, and *IGL* genes. It is likely that these genes and other non-rearranging genes encoding immune-related cell surface proteins share a common origin[71]. For instance, the rodent *Cd8b* genes encode a single V-like domain followed by a J-like sequence (**FG**T**G**TKLTVG; canonical FGXG signature highlighted), which is remarkably similar to the J elements of *IgL* and T*RA/TRB* loci; however, unlike the situation in AgR, there is no intervening sequence between the V-like and J-like sequences[72].

For the present study, we focused on *TRA* genes for the following reasons.
(1) The *TRG* locus often consists of only very few *V* and *J* elements. For instance, in the mouse, 7 *Vg* and 4 *Jg* elements are recognized; in the zebrafish, 7 *Vg* and 7 *Jg* are known. By contrast, the assemblies arising from the *TRA* locus are much more complex, usually involving dozens of *V* and *J* elements (mouse: 64 *Va* and 45 *Ja* elements respectively; zebrafish: 124 *Va* and 134 *Ja* elements respectively). Based on the data deposited in the IMGT database (https://www.imgt.org/), differences in the numbers of *V* and especially *J* segments between *TRG* and *TRA* also hold for 11 other species (Supplementary Figure 1a).
(2) It has now been shown for many species (such as squamates[24]) that they have lost the genes for the TCRγδ receptor. Apart from introducing bias in broad phylogenetic surveys, the statistical robustness of any conclusion about conserved sequence elements is inversely proportional to the numbers *V* and *J* elements that are associated with *TRG*. Hence, we consider the inferences derived from the analyses of *TRA* loci to be less prone to random fluctuations and therefore more meaningful.
(3) *IgL* loci commonly present with few *J* elements, although the number of *V* elements varies considerably (Supplementary Fig. 1b). By way of example, a total of 51 V_L genes and 17 J_L (all 5 loci combined) were described for zebrafish[73], and 146 V_L and 12 J_L for mouse (κ and λ loci combined; https://www.imgt.org/).
(4) Since the numbers and structures of *IgL* gene loci vary among vertebrate species, it is not straightforward to establish orthology, complicating inferences based on phylogenetic relationships.

In Supplementary Figures 2-5, we provide information on the structure of *V* and *J* elements of *TRG* and *IGL* loci in zebrafish and mouse, and identify conserved sequence patterns at the ends of *Vg* and *Jg* elements. This analysis described below suggests that microhomology-directed recombination is a general feature of V-J type antigen receptor loci, although it is most easily recognizable for the *TRA* genes.
<u>*TRG* loci:</u>
(1) The positions of the RSS relative to the conserved cysteine codon in *Vg* elements of zebrafish vary (Supplementary Fig. 2), in stark contrast to the situation of *Va* elements in this species (Extended Data Fig. 1). In contrast to the situation of *Ja* of zebrafish, the *Jg* elements lack a consistent sequence motif downstream of the RSS elements (Supplementary Fig. 2). This leads to a much greater length variation of the CDR3 regions of *TRG* assemblies (Supplementary Fig. 2), in contrast to the stereotyped 3-nucleotide step pattern observed in *tra* assemblies (Extended Data Figure 3). The sequence analysis of the *trg* assemblies does however suggest the possibility of occasional microhomology-directed recombination, although this phenomenon is less obvious than in the *tra* assemblies, at least based on the few zebrafish *trg* assemblies that have been reported (Supplementary Fig. 2).
(2) For the mouse *Trg* elements, the structure of the germ-line *Vg* and *Jg* elements also suggests the possibility of microhomology-directed recombination (Supplementary Fig. 3), which has already been experimentally demonstrated[32].

<u>*IGL* loci</u>:
(1) Sequence alignments of V_L and J_L elements for the five genomic clusters described by Zimmerman et al.[73], (Supplementary Fig. 4) indicate the presence of similar sequence motifs at the ends of *V* and *J* elements for some, but not all clusters.
(2) For the mouse (Supplementary Fig. 5), it appears that matching sequence motifs are also detectable, but they are not conserved across different species.
<u>*TRD* loci</u>:
By way of comparison, we also analysed the *trd* locus of a teleost to examine whether the V-D-J configuration of AgR genes, which is considered to be a derived form of the primordial V-J configuration[1,17] (for an alternative evolutionary scenario, see[74]) retains an appreciable degree of microhomology.
In Supplementary Figure 7, we provide an analysis of the sequence signatures of *Vd*, *Dd* and *Jd* elements of *P. progenetica*[40]. The *V* elements dominating in *trd* assemblies (6/54 in the *Va/Vd* cluster) are almost never found in *tra* assemblies. Moreover, they exhibit no recognizable shared sequence pattern next to the RSS, clearly distinguishing them from a typical *Va* element. We note that the two *Jd* elements also do not share sequence similarity next to the RSS, nor to the ends of the *Vd* or *Dd* segments. These results suggest that, although *Vd* elements are scattered across the *Va/Vd* cluster, they lack the 'primordial' signatures, in line with the fact that *trd* assemblies not only incorporate *D* elements, but also exhibit a significant number of non-templated nucleotides as described in our previous study[40].
In view of the above summary, we considered the *TRA* locus to be the best target for our investigation, as it is a constant companion of canonical adaptive immune systems in jawed vertebrates (with the exception of a small number of species of deep-sea anglerfishes that we recently found to have lost canonical adaptive immunity after pseudogenization of RAG genes[23]).

The role of TdT in diversification of CDR3 regions

TdT is a vertebrate-specific paralogue of DNA polymerase μ[75] and is capable of catalyzing the addition of nucleotides to the 3' terminus of a DNA molecule without requiring a template. TdT contributes to the diversification of coding joints in the V(D)J recombination process[34-36]; its activity is strictly regulated during lymphocyte development[76], as reflected in the variable numbers of non-templated nucleotides in CDR3 regions of *AgR* assemblies. For zebrafish, it has been shown that TdT is expressed in the cortex of the thymus[37], and is differentially expressed in various subsets of thymocytes[38], as determined by RNA *in situ* hybridization and scRNA-seq respectively. These observations suggest that the developmental regulation of TdT may be a conserved, yet evolutionarily malleable, feature of lymphocyte development.
Of note, a functional entanglement exists between the presence of microhomology regions at the ends of *V* and *J* elements and the presence or absence of TdT activity during recombination. TdT activity impairs the use of microhomologies during the NHEJ-mediated repair process[34,35]. Therefore, microhomology regions will likely degrade over evolutionary time, if recombination at a particular locus always occurs in the presence of TdT.

References

71. Johnson, P. & Williams, A. F. Striking similarities between antigen receptor J pieces and sequence in the second chain of the murine CD8 antigen. *Nature* **323**, 74-76 (1986).
72. Hunkapiller. T & Hood, L. The growing immunoglobulin gene superfamily. *Nature* **323**, 15-16 (1986).
73. Zimmerman, A. M., Yeo, G., Howe, K., Maddox, B. J. & Steiner, L. A. Immunoglobulin light chain (IgL) genes in zebrafish: Genomic configurations and inversional rearrangements between (V(L)-J(L)-C(L)) gene clusters. *Dev. Comp. Immunol.* **32**, 421-434 (2007).
74. Siu, G., Kronenberg, M., Strauss, E., Haars, R., Mak, T.W. & Hood, L. The structure, rearrangement and expression of D beta gene segments of the murine T-cell antigen receptor. *Nature* **311**, 344-350 (1984).
75. Bart, S. et al. Terminal deoxynucleotidyl transferases from elasmobranchs reveal structural conservation within vertebrates. *Immunogenetics* **55**, 594-604 (2003).
76. Coleman, M. S., Yang, B. & Sorscher, D. Regulation of terminal deoxynucleotidyl transferase gene expression in mice and men. Crit. Rev. *Eukaryot. Gene Expr.* **2**, 237-250 (1992).

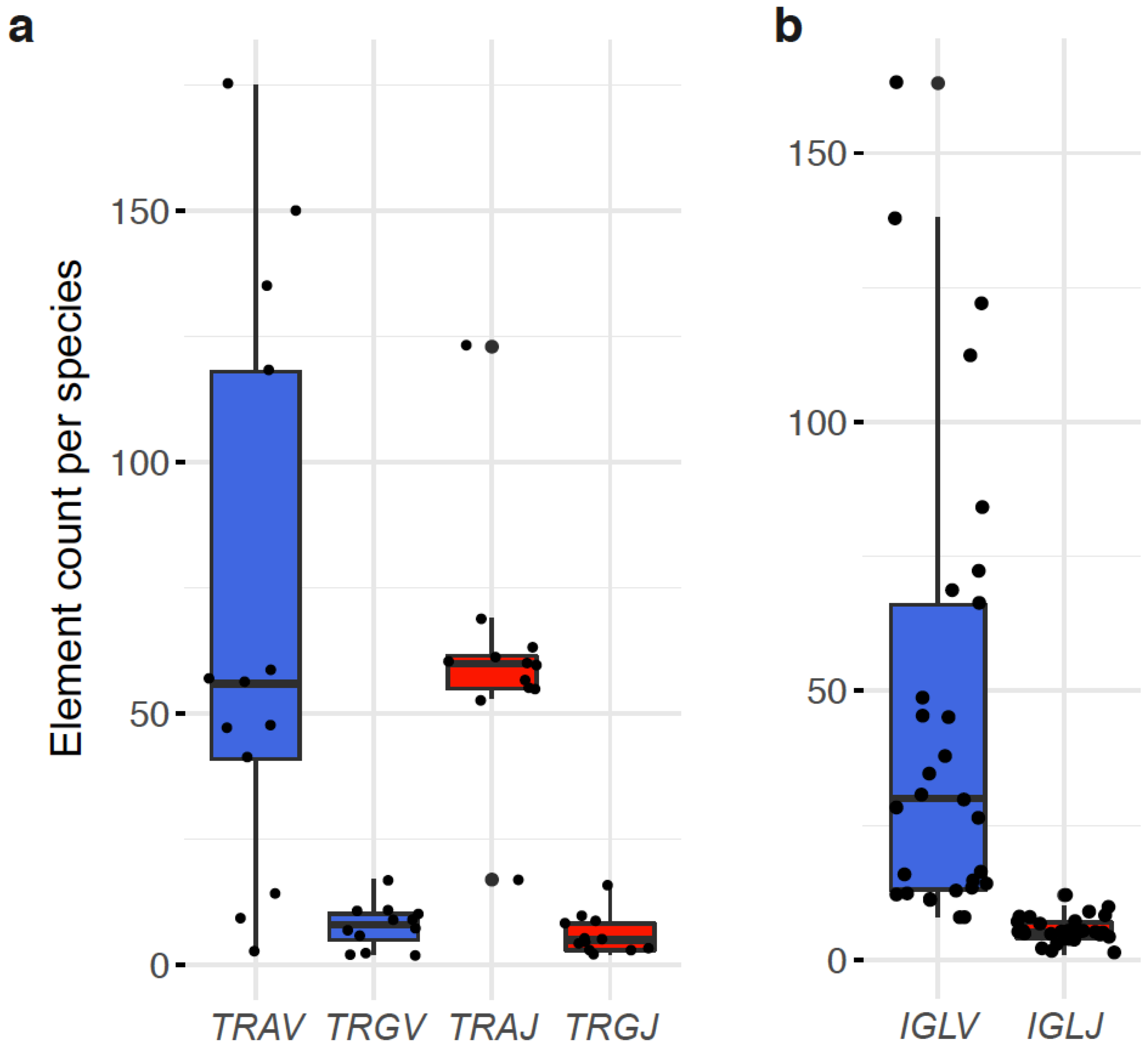

Supplementary Figure 1 | Species-specific differences in the number of V and J elements for three types of V-J antigen receptor genes. a, Number of *V* and *J* elements for T cell receptor alpha (*TRA*) and T cell receptor gamma (*TRG*) loci of species listed in the IMGT database. **b**, Number of *V* and *J* elements for immunoglobulin light chain gene (*IGL*) loci of species listed in the IMGT database. For **a** and **b**, each dot represents a single species. Shown are the median (horizontal line), the first and third quartile (box); the wiskers are drawn for a distance of 1.5 times the interquartile range up to the largest/lowest data point from the dataset that falls within this distance.

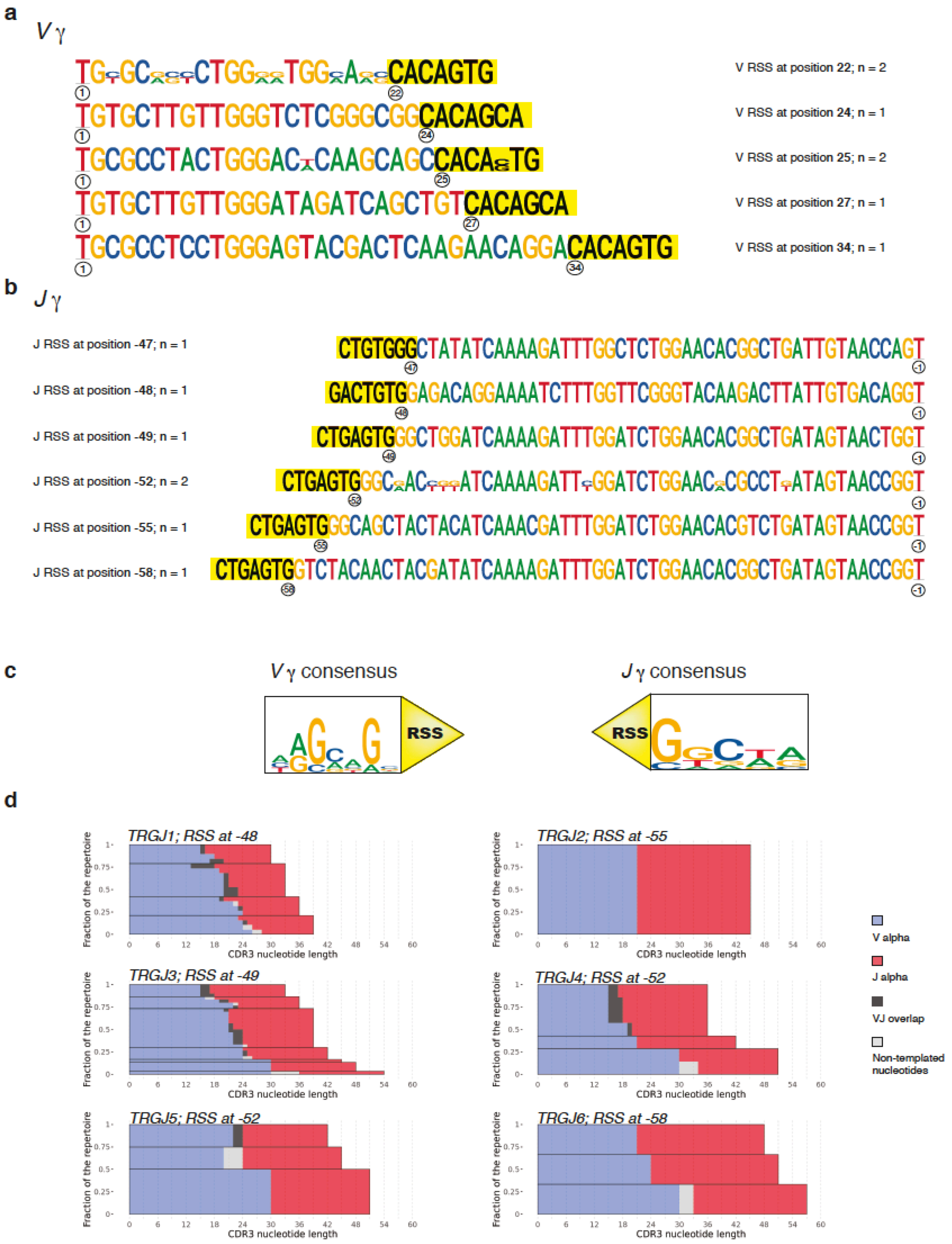
a
Vγ
V RSS at position 22; n = 2
V RSS at position 24; n = 1
V RSS at position 25; n = 2
V RSS at position 27; n = 1
V RSS at position 34; n = 1
b
Jγ
J RSS at position -47; n = 1
J RSS at position -48; n = 1
J RSS at position -49; n = 1
J RSS at position -52; n = 2
J RSS at position -55; n = 1
J RSS at position -58; n = 1
c
Vγ consensus
RSS
Jγ consensus
RSS
d
TRGJ1; RSS at -48
TRGJ2; RSS at -55
TRGJ3; RSS at -49
TRGJ4; RSS at -52
TRGJ5; RSS at -52
TRGJ6; RSS at -58
Fraction of the repertoire
CDR3 nucleotide length
V alpha
J alpha
VJ overlap
Non-templated nucleotides

Supplementary Figure 2 | Characterization of the T cell receptor gamma locus of zebrafish. **a,** Genomic sequences of *Vg* elements. Position number +1 corresponds to the first nucleotide in the characteristic cysteine codon at the 3´end of *V* elements. The heptamer sequences are highlighted in yellow, the position of the first base of the heptamer is indicated on the right to the sequences, as is the number of *V* elements conforming to this position. **b**, Genomic sequences of *Jg* elements. Position number –1 corresponds to the thymidine of the GT splice donor site at the 3´-end of *J* elements. The heptamer sequences are highlighted in yellow, the positions of the first base of the heptamers are indicated on the left of the sequences, as are the number of *J* elements conforming to this position. **c**, Consensus sequences of the last 7 (*Vg*) and first 5 (*Jg*) sequences, situated 5´- (*Vg*) or 3´- (*Jg*) of the RSS sequences. **d**, Schematic representation of the *trg* assemblies of *D. rerio* taken from Genbank accession numbers AY973880.1 to AY973943.1, grouped according to the *J* element used. *VJ* overlaps are indicative of microhomology-mediated recombination.

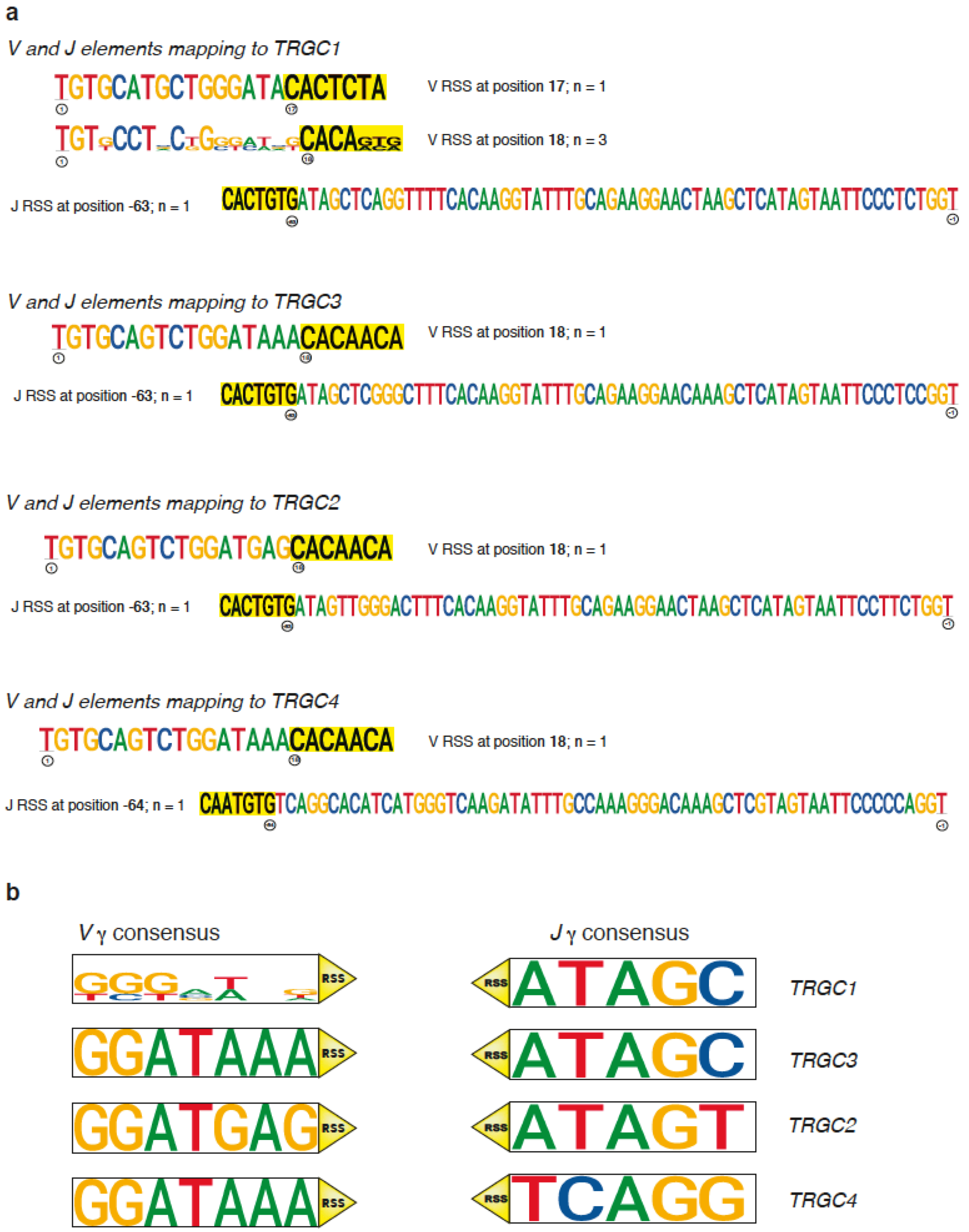
a
V and J elements mapping to TRGC1
TGTGCATGCTGGGATACACTCTA
V RSS at position 17; n = 1
V RSS at position 18; n = 3
J RSS at position -63; n = 1
CACTGTGATAGCTCAGGTTTTCACAAGGTATTTGCAGAAGGAACTAAGCTCATAGTAATTCCCTCTGGT
V and J elements mapping to TRGC3
TGTGCAGTCTGGATAAACACAACA
V RSS at position 18; n = 1
J RSS at position -63; n = 1
CACTGTGATAGCTCGGGCTTTCACAAGGTATTTGCAGAAGGAACAAAGCTCATAGTAATTCCCTCCGGT
V and J elements mapping to TRGC2
TGTGCAGTCTGGATGAGCACAACA
V RSS at position 18; n = 1
J RSS at position -63; n = 1
CACTGTGATAGTTGGGACTTTCACAAGGTATTTGCAGAAGGAACTAAGCTCATAGTAATTCCTTCTGGT
V and J elements mapping to TRGC4
TGTGCAGTCTGGATAAACACAACA
V RSS at position 18; n = 1
J RSS at position -64; n = 1
CAATGTGTCAGGCACATCATGGGTCAAGATATTTGCCAAAGGGACAAAGCTCGTAGTAATTCCCCCAGGT
b
Vγ consensus
Jγ consensus
RSS
GGATAAA
GGATGAG
GGATAAA
ATAGC
ATAGC
ATAGT
TCAGG
TRGC1
TRGC3
TRGC2
TRGC4

Supplementary Figure 3 | Characterization of the T cell receptor gamma locus of mouse. a, Partial nucleotide sequences of *Vg* and *Jg* elements grouped by the four known loci. For *V* elements, position number +1 corresponds to the first nucleotide in the characteristic cysteine codon at the 3′end of *V* elements. For *J* elements, position number –1 corresponds to the thymidine of the GT splice donor site at the 3′-end of *J* elements. The heptamer sequences are highlighted in yellow, with the positions of the first base of the heptamers indicated. The number of *V* and *J* elements conforming to a certain position of RSS is also indicated. **b**, Consensus nucleotide sequences of the last 7 (*Vg*) and first 5 (*Jg*) sequences situated 5′- (*Vg*) or 3′- (*Jg*) of the RSS sequences, grouped according to cluster.

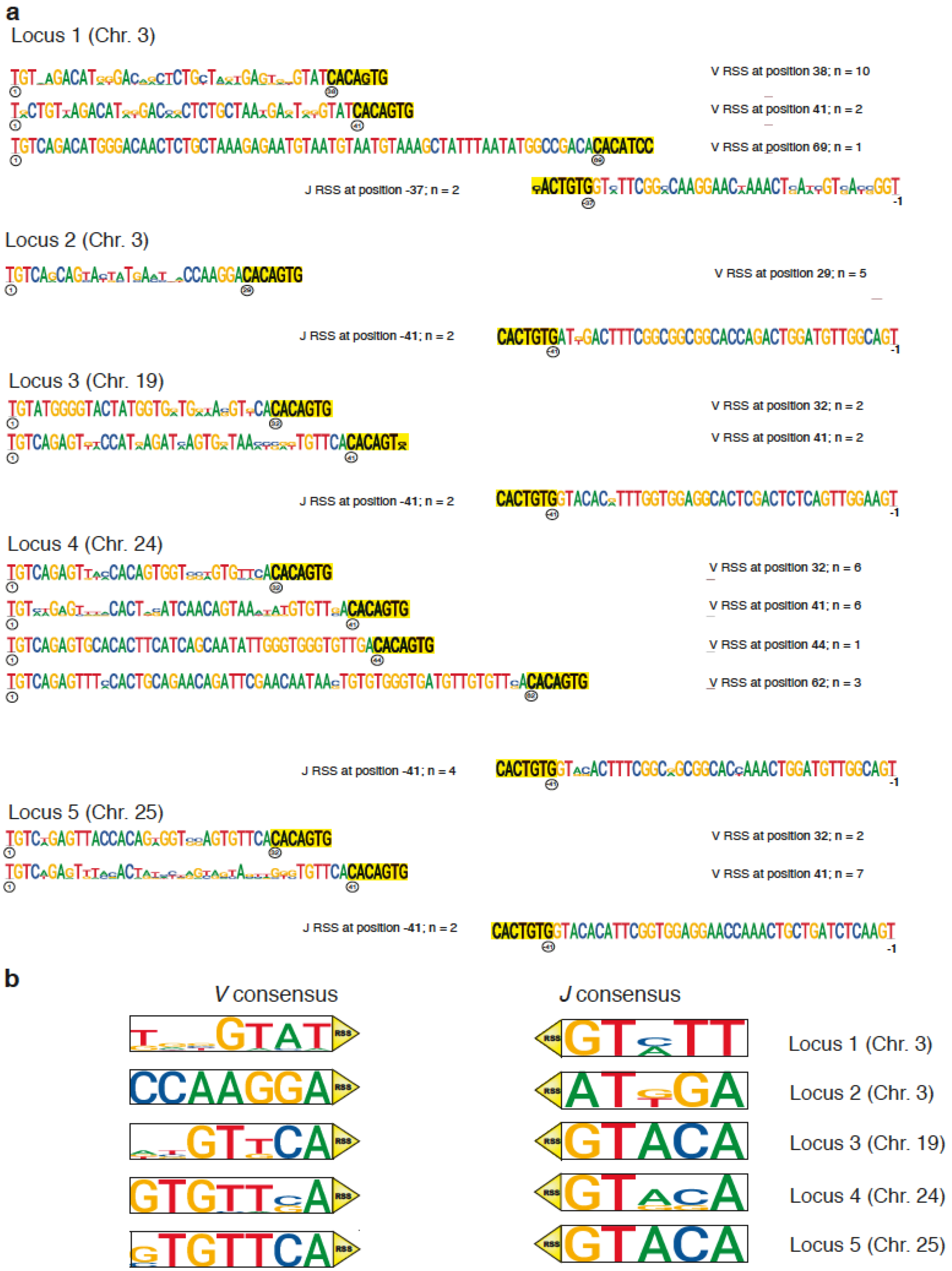
a
Locus 1 (Chr. 3)
V RSS at position 38; n = 10
V RSS at position 41; n = 2
V RSS at position 69; n = 1
J RSS at position -37; n = 2
Locus 2 (Chr. 3)
V RSS at position 29; n = 5
J RSS at position -41; n = 2
Locus 3 (Chr. 19)
V RSS at position 32; n = 2
V RSS at position 41; n = 2
J RSS at position -41; n = 2
Locus 4 (Chr. 24)
V RSS at position 32; n = 6
V RSS at position 41; n = 6
V RSS at position 44; n = 1
V RSS at position 62; n = 3
J RSS at position -41; n = 4
Locus 5 (Chr. 25)
V RSS at position 32; n = 2
V RSS at position 41; n = 7
J RSS at position -41; n = 2
b
V consensus
J consensus
Locus 1 (Chr. 3)
Locus 2 (Chr. 3)
Locus 3 (Chr. 19)
Locus 4 (Chr. 24)
Locus 5 (Chr. 25)

Supplementary Figure 4 | Characterization of immunoglobulin light chain loci of zebrafish. **a,** Partial nucleotide sequences of V_L and J_L elements grouped by the five known loci; the chromosomal localizations are indicated. For *V* elements, position number +1 corresponds to the first nucleotide in the characteristic cysteine codon at the 3′end of *V* elements. For *J* elements, position number –1 corresponds to the thymidine of the GT splice donor site at the 3′-end of *J* elements. The heptamer sequences are highlighted in yellow, with the position of the first base of the heptamers indicated. The number of *V* and *J* elements conforming to a certain position of RSS is also indicated. **b**, Consensus nucleotide sequences of the last 7 (V_L) and first 5 (J_L) sequences situated 5′- (V_L) or 3′- (J_L) of the RSS sequences, grouped according to cluster.

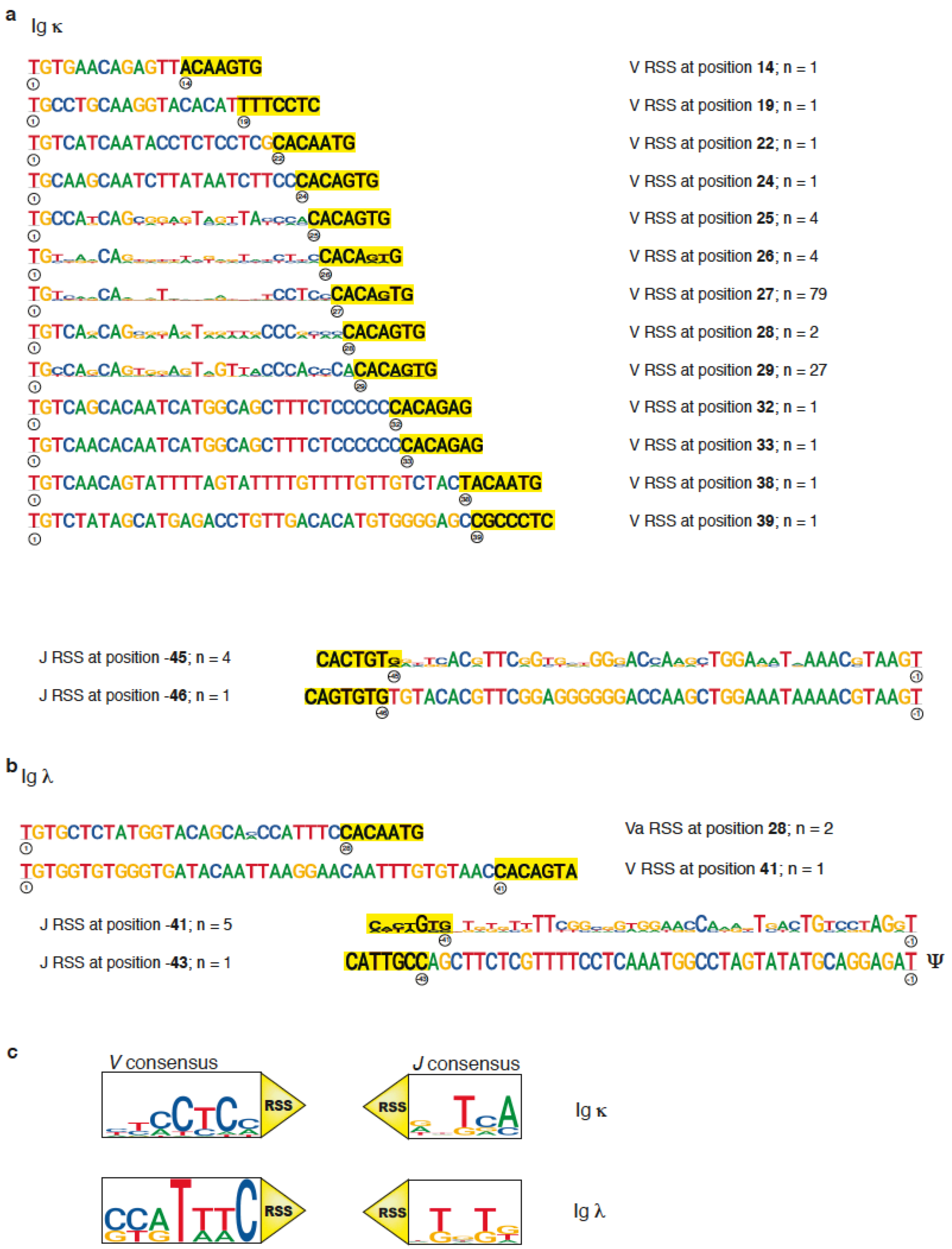
a
Ig κ
TGTGAACAGAGTTACAAGTG
V RSS at position 14; n = 1
TGCCTGCAAGGTACACATTTTCCTC
V RSS at position 19; n = 1
TGTCATCAATACCTCTCCTCGCACAATG
V RSS at position 22; n = 1
TGCAAGCAATCTTATAATCTTCCCACAGTG
V RSS at position 24; n = 1
V RSS at position 25; n = 4
V RSS at position 26; n = 4
V RSS at position 27; n = 79
V RSS at position 28; n = 2
V RSS at position 29; n = 27
TGTCAGCACAATCATGGCAGCTTTCTCCCCCCACAGAG
V RSS at position 32; n = 1
TGTCAACACAATCATGGCAGCTTTCTCCCCCCCACAGAG
V RSS at position 33; n = 1
TGTCAACAGTATTTTAGTATTTTGTTTTGTTGTCTACTACAATG
V RSS at position 38; n = 1
TGTCTATAGCATGAGACCTGTTGACACATGTGGGGAGCCGCCCTC
V RSS at position 39; n = 1
J RSS at position -45; n = 4
J RSS at position -46; n = 1
CAGTGTGTGTACACGTTCGGAGGGGGGACCAAGCTGGAAATAAAACGTAAGT
b Ig λ
TGTGCTCTATGGTACAGCA CCATTTCCACAATG
Va RSS at position 28; n = 2
TGTGGTGTGGGTGATACAATTAAGGAACAATTTGTGTAACCACAGTA
V RSS at position 41; n = 1
J RSS at position -41; n = 5
J RSS at position -43; n = 1
CATTGCCAGCTTCTCGTTTTCCTCAAATGGCCTAGTATATGCAGGAGAT Ψ
c
V consensus
J consensus
RSS
Ig κ
Ig λ

Supplementary Figure 5 | Characterization of immunoglobulin light chain loci of mouse. a, Partial nucleotide sequences of V_L and J_L elements in the kappa locus. For *V* elements, position number +1 corresponds to the first nucleotide in the characteristic cysteine codon at the 3′end of *V* elements. For *J* elements, position number –1 corresponds to the thymidine of the GT splice donor site at the 3′-end of *J* elements. The heptamer sequences are highlighted in yellow, with the position of the first base of the heptamer indicated. The number of *V* and *J* elements conforming to a certain position of RSS is also indicated. **b**, Partial nucleotide sequences of V_L and J_L elements in the lambda locus. For *V* elements, position number +1 corresponds to the first nucleotide in the characteristic cystein codon at the 3′end of *V* elements. For *J* elements, position number –1 corresponds to the thymidine of the GT splice donor site at the 3′-end of *J* elements (note that one J element is considered a pseudogene (Ψ), since the splice donor site is mutated (GT>AT). The heptamer sequences are highlighted in yellow, with the position of the first base of the heptamers indicated. The number of *V* and *J* elements conforming to a certain position of RSS is also indicated**. c**, Consensus nucleotide sequences of the last 7 (V_L) and first 5 (J_L) sequences situated 5′- (V_L) or 3′- (J_L) of the RSS sequences, grouped according to cluster.

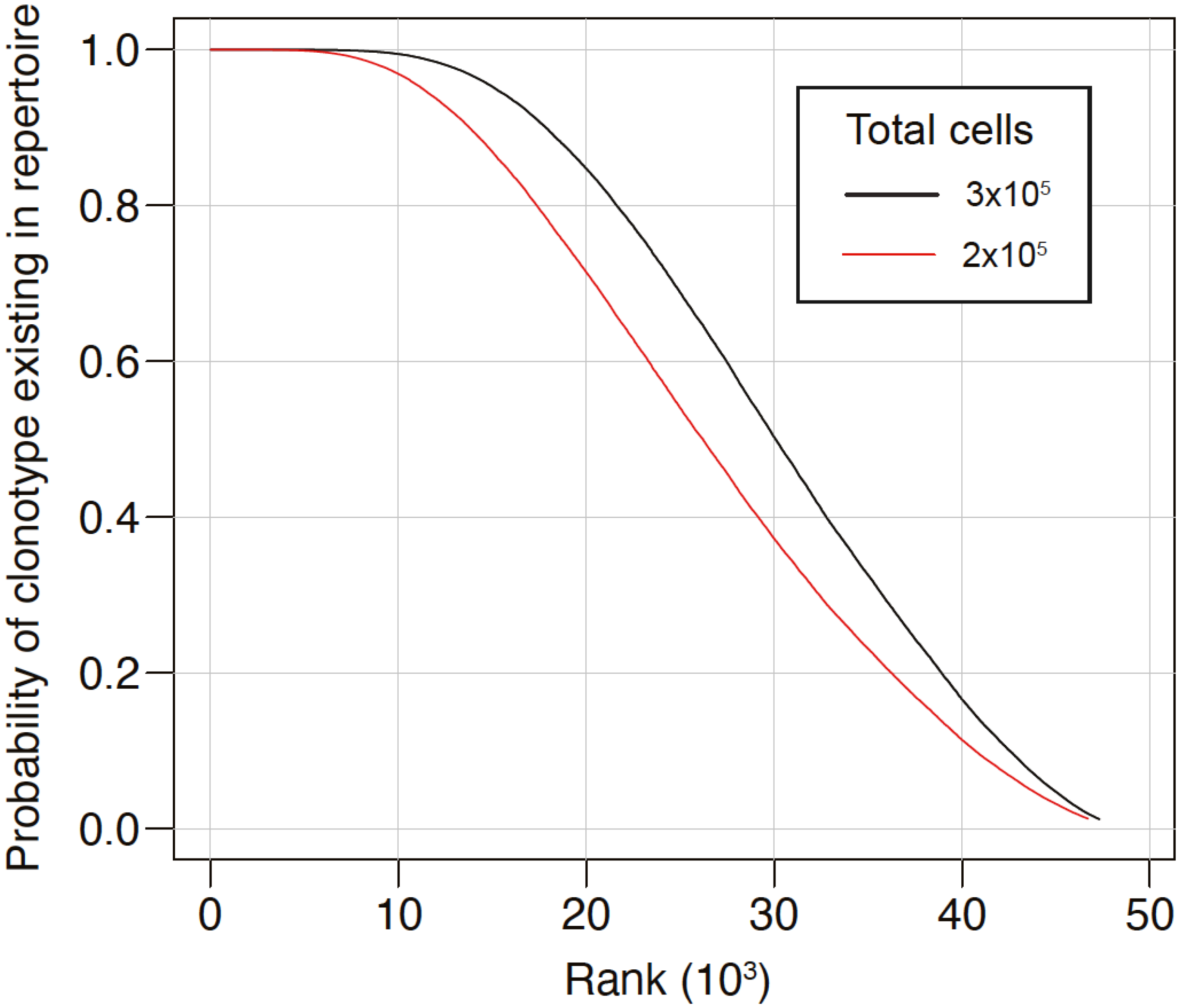

Supplementary Figure 6 | Predictability of *tra* repertoire in *D. rerio*. The probability of generation of a particular clonotype can be deduced from the combination of *V* and *J* elements and the effect of microhomology-directed recombination. The results are shown for lymphoid compartments of 200,000 and 300,000 T cells, which is within the range estimated for adult zebrafish (see Methods).

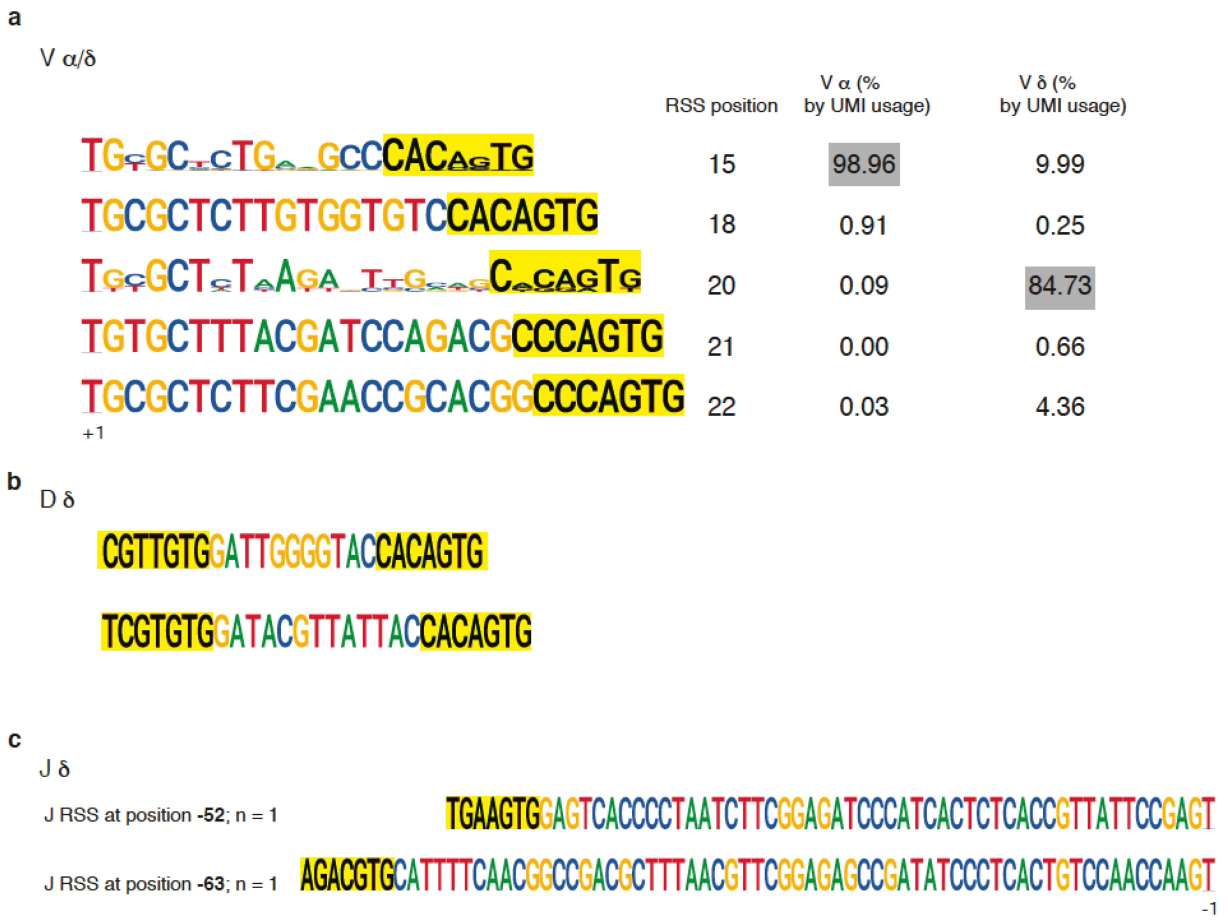

Supplementary Figure 7 | Analysis of the TCR alpha/delta locus in *P. progenetica* (minifish). **a**, Characterization of *V* elements grouped by the positions of the RSS elements (highlighted in yellow). The first nucleotide shown corresponds to the first nucleotide in the characteristic cysteine codon at the 3´end of *V* elements. In the right-hand colums, the usage of *V* elements as per position of RSS is indicated (see also Extended Data Figure 1). Note that *V* elements with RSS at position 20 are almost exclusively used in TCR delta assemblies. **b**, Characterization of the two *D* delta elements; the heptamer sequences are highlighted in yellow. **c**, Characterization of the two *J* delta elements; nucleotide position number –1 corresponds to the thymidine of the GT splice donor site at the 3´-end of *J* elements and heptamer sequences are highlighted in yellow.

Legends to Supplementary Tables

Supplementary Table 1 | Repertoire data statistics on sequences with mapped *V* and *J* elements. UMIs (cDNA molecules) and clone counts exclude valid rearrangements from genes not found in the available assemblies (see also Supplementary Tables 2, 3). The proportion of mapped to total valid rearrangements is displayed for *V*, *J* and *VJ*: all proportions are weighted in UMI usage. *VJ* combinations represents the count of identifiable pairs of gene elements. Values for mean length of CDR3 sequences (nucleotides), the proportion of in-frame CDR3s, and the numbers of excised nucleotides from *VJ*, *VD* or *DJ* joints are also weighted. The values for *VD* and *DJ* joints were calculated only for sequences where a *D* segment of 5 nucleotides or longer could be identified. For *TRA* assemblies, the column labelled "no *VJ* insertions" includes both microhomology and blunt end joints. Entropy is estimated in bits (see Methods), without weighting. The location of RSS relative to reading frame (see Fig. 2a) was weighted by usage.

Supplementary Table 2 | Genomic locus descriptions for *TRA* genes of species whose repertoires were analyzed in this study. Accession numbers are provided together with individual positions of genes. Note that some genomes are not available as assembled chromosomes and a concatenation of scaffolds was used, without attempting to reconstruct their true order. For the genome of *C. punctatum,* three scaffolds were concatenated as follows: ccg_chipu00000448–ccg_chipu00000257–ccg_chipu00000318. For the genome of *L. africana*, scaffolds were concatenated as follows: GL010131.1(nucleotides 1–2,000,000 in forward direction)–GL010145.1(nucleotides 1–1,000,000 in reverse direction)–GL010280.1(in forward direction)–GL010422.1(in forward direction)–GL010594.1(in reverse direction)–AAGU03094560.1(reverse direction)–AAGU03094287.1(forward direction). F, transcribed functional element; ORF, functional element without evidence of transcription; P, pseudogenized element. The symbol "0" denotes *V* elements for which the positions of the intron could not be unambiguously defined; note that for Dβ, Jα/Jβ elements, and the first exons of Cα/Cβ genes, no intron is defined.

Supplementary Table 3 | Genomic locus descriptions for *TRB* genes of species whose repertoires were analyzed in this study. Accession numbers are provided together with individual positions of genes. For the genome of *L. africana*, the *TRB* locus occurs on three scaffolds, which were concatenated as follows: GL010110.1(nucleotides 1 – 1,000,000)–GL010632.1–AAGU03094591.1; no attempt was made to determine the true order of scaffolds in the genome. F, transcribed functional element; ORF, functional element without evidence of transcription; P, pseudogenized element.

Supplementary Table 4 | Germline elements mapping to *Ja* regions in genomes of species analyzed in this study. Number of *J* elements (see Methods) identified between and including the first and last J elements in the *Ja* regions. Entropy of the first 5 positions after RSS (see Fig. 4b). Proportion of *Ja* elements exhibiting the conserved teleost position of RSS, when analyzed

using zebrafish and mouse query algorithms (see Methods). Accession numbers and identifiers are provided for each species. When NA values are assigned to a particular species, this species is not included in the analysis, because either *TRAC* or *TRDC* elements were not found in the *Ja*-containing scaffold. File name; automatically generated identifier for exported sequences.

Supplementary Table 5 | Summary of sequence runs. Machine settings and threshold reads used in the analysis are displayed for individual runs. Statistics of the number of runs, including mapped total numbers and amount of errors detected by clustering (see Methods) are detailed. Accession numbers for raw data are provided.

Supplementary Table 6 | Oligonucleotide primers used in this study.
